# Case Report: Extended cardiopulmonary resuscitation in sudden cardiac arrest after acute myocardial infarction

**DOI:** 10.3389/fcvm.2024.1412104

**Published:** 2024-08-09

**Authors:** Zhongkai Yu, Yubin Hu, Xiuli Chen

**Affiliations:** ^1^ Department of Emergency, Liaocheng People’s Hospital, Liaocheng, Shandong, China; ^2^ Department of Internal Medicine, People’s Hospital of Qingyun County, Dezhou, Shandong, China

**Keywords:** cardiac arrest, ECMO, acute myocardial infarction, causes of disease, closed chest cardiac massage

## Abstract

Out-of-hospital cardiac arrest (OHCA) mostly occurs in crowded public places outside hospitals, such as public sports facilities, airports, railway stations, subway stations, and shopping malls. The emergency department of Liaocheng People's Hospital in Shandong Province admitted one patient with OHCA in August 2021, who suddenly suffered a loss of consciousness and cardiac arrest during exercise after dinner. Witnesses immediately gave continuous chest compressions and artificial respiration and called our hospital's emergency department (at 120). Arriving at the emergency department, we continued to provide chest compressions and ventilator-assisted ventilation after performing endotracheal intubation. We administered adrenaline for cardiac excitation, dopamine for maintained blood pressure, sodium bicarbonate to correct the acidosis, and multiple electric defibrillations. However, the patient's cardiac Doppler ultrasound indicated poor cardiac contractions, and extracorporeal membrane oxygenation (ECMO) was started immediately. We performed coronary angiography for the patient with ECMO support, indicating that the patient had an 80% critical stenosis of the left main coronary artery and an 80%–90% stenosis in the middle section of the left anterior descending artery with an aneurysm. Fortunately, there was no obvious stenosis in the right coronary artery. The patient was transferred to the intensive care unit and received comprehensive treatment, including anticoagulation, myocardial nutritional support, improvement of cardiac function, continuous renal replacement therapy, organ function protection, anti-inflammatory treatment, and rehabilitation. Coronary artery bypass grafting was performed after the patient's condition stabilized, and he was finally discharged. ECMO support therapy for patients with cardiac arrest can be considered when economic conditions permit. It is very important to conduct the necessary examinations in the early stage of resuscitation with ECMO support to clarify the cause of the cardiac arrest and to treat it accordingly.

## 1 Introduction

Cardiac arrest (CA) has become a major public health problem. In the United States, more than 350,000 people experience out-of-hospital CA (OHCA), and over 290,000 people in-hospital CA (IHCA) every year (1, 2). In China, the incidence of CA is about 600,000 a year (3), and the overall prognosis of patients with CA is not ideal. The survival rates for IHCA and OHCA are 25%–30% and 10%–15%, respectively. In recent years, extracorporeal cardiopulmonary resuscitation (ECPR) has rapidly developed, and its application in the treatment of CA has gradually attracted attention (4). ECPR refers to cardiopulmonary resuscitation for patients with CA who do not respond to conventional cardiopulmonary resuscitation (CCPR) supported by extracorporeal membrane oxygenation (ECMO). ECMO can provide circulatory and respiratory support for patients quickly, ensuring the perfusion of important organs such as the heart and brain, promoting the recovery of autonomous circulation following CA, and avoiding hypoxic damage in order to improve survival rates and neurological prognoses (5).

## 2 Case description

**Figure 1:**
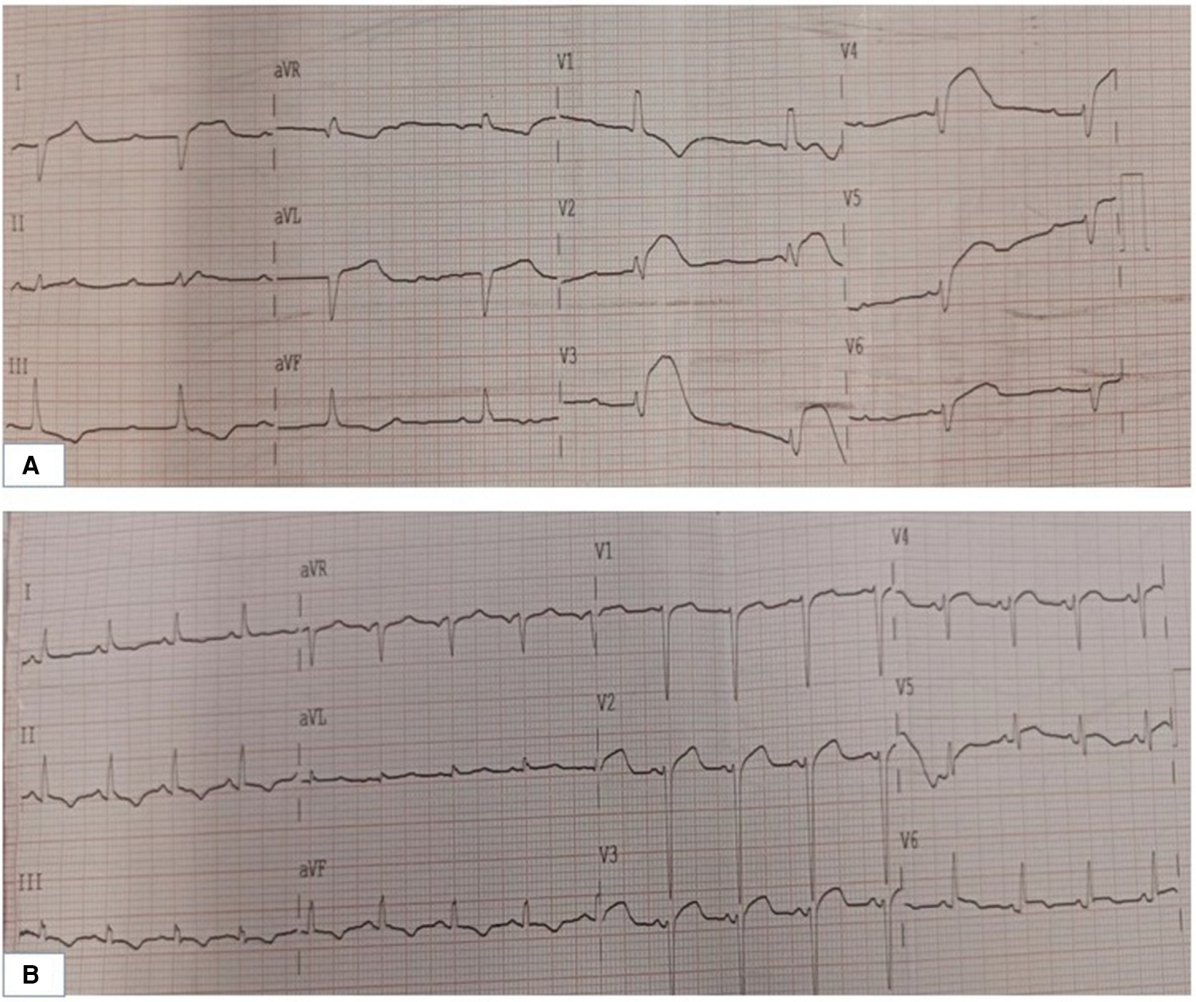
(**A**) Electrocardiogram of the patient during the rescue. (**B**) Electrocardiogram of the patient following the ECMO. ECMO: extracorporeal membrane oxygenation.

**Figure 2:**
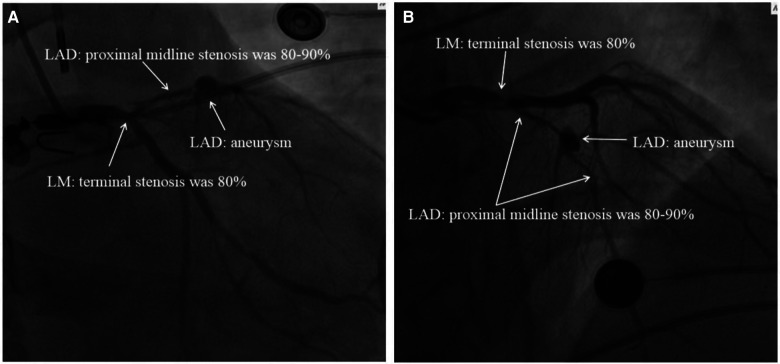
Images of the patient's cardiogram. (**A**) Foot position angle imaging. (**B**) Head position angle imaging: the end of the LM in this patient had 80% stenosis, and the proximal and middle segments of the LAD had 80%–90% stenosis accompanied by an aneurysm. LM, left main coronary artery; LAD, left anterior descending branch.

A 36-year-old male patient with a history of alcoholism (500 ml liquor a day) and smoking (20 cigarettes a day) suddenly became unconscious and experienced CA at 19:34 on August 17, 2021, while he was exercising in the street after dinner. Witnesses gave the patient extrathoracic cardiac compressions and artificial respiration immediately and called 120 to transport the patient to our hospital. At 20:10, the patient was admitted to the Emergency Department of Liaocheng People's Hospital. At this time, we found that the patient was apneic and pulseless, with no recordable blood pressure, consciousness or bilateral pupillary light reflexes, and facial cyanosis. The patient's Glasgow Coma Scale (GCS) score was only 3 (1-1-1). Meanwhile, we performed endotracheal intubation and started ventilator-assisted ventilation immediately. Color Doppler echocardiography examination indicated that the patient's heart had no contractile activity. Blood gas analysis showed the following: PH of 7.06, PO_2_ of 22 mmHg, PCO_2_ of 64 mmHg, sodium of 145 mmol/L, potassium of 4.2 mmol/L, lactic acid of 10.7 mmol/L, and HCO_3_- of 12.1 mmol/L. The formal laboratory examination results are shown in Table 1. We immediately gave the patient adrenaline injections (1 mg/3 min) to stimulate the heart, dopamine to maintain the blood pressure, and sodium bicarbonate to correct the acidosis. When the electrocardiographic monitoring indicated repeated ventricular fibrillation, the patient was given multiple electrical defibrillations until the spontaneous rhythm was restored (Figure 1). However, the cardiac color Doppler ultrasound showed that the cardiac ejection fraction (EF) was only 20%, so ECMO was immediately started to support the heart with the consent of the patient's family. Veno-arterial ECMO (VA-ECMO) was initiated with right lower limb femoral vein catheterization for blood drainage (21 F venous cannula) and left lower limb femoral artery catheterization for blood transfusion after membrane oxygenation (15 F femoral arterial cannula). The ECMO blood flow was 3,000 L/min. Meanwhile, a distal limb perfusion cannula was inserted. Under VA-ECMO support, coronary angiography was performed. We found that the end of the left main coronary artery was 80% stenosed, and the proximal and middle segments of the left anterior descending artery had 80%–90% stenosis, accompanied by an aneurysm (Figure 2). Due to the patient's serious condition, the diseased coronary artery was balloon-dilated as a temporary measure, balloon vasodilation temporarily relieved the stenosis of the patient's coronary artery, thus enabling the diseased coronary blood flow to be unobstructed and allowing TIMI flow to approach grade 3. The patient was then transferred to the intensive care unit (ICU).

**Table 1:** Laboratory examination results.

	Emergency tests	ICU tests					General ward tests	
	8.17	8.18	8.19	8.21	8.26	8.31	9.6	9.10
WBC	13.69	12.01	13.44	12.05	11.06	10.9	6.85	7.93
RBC	4.70	4.43	4.66	4.03	4.23	3.26	4.50	4.35
Hb	136	128	136	117	125	108	126	132
PLT	166	147	126	120	110	249	211	179
NEUT	5.39	10.61	12.4	10.71	8.79	8.45	5.15	5.34
NEUT (%)	39.4	88.3	92.2	88.8	79.4	77.5	75.2	67.4
CRP	0.03	50.91	38.99	29.79	32.36	16.3	10.76	2.65
ALT	40	150	119	43	45	39	32	27
AST	36	525	279	37	39	36	32	28
Scr	84.6	125.0	84.4	75.9	58.1	62.9	48.1	52.5
BUN	5.88	9.07	5.02	7.60	7.12	7.63	5.48	3.63
BNP	83	2,050	3,230	2,520	1,860	1,150	378	125
cTn I	13.15	85	25.95	10.38	2.19	0.26	<0.01	<0.01
CKMB	19.21	250.76	58.42	1.55	1.77	1.14	0.92	1.06

ICU, intensive care unit; WBC, white blood cell count (normal range is 3.5 × 10^9^/L to 9.5 × 10^9^/L); RBC, red blood cell count (normal range is 4.5 × 10^12^/L to 5.5 × 10^12^/L); Hb, hemoglobin (normal range is 130 g/L to 175 g/L); PLT, platelet count (normal range is 100 × 10^9^/L to 300 × 10^9^/L); NEUT, neutrophil count (normal range is 2.0 × 10^9^/L to 6.0 × 10^9^/L); NEUT%: neutrophil ratio (normal range is 40% to 75%); CRP, C-reactive protein (normal range is 0 mg/L to 10 mg/L); ALT, alanine aminotransferase (normal range is 10 U/L to 50 U/L); AST, aspartate aminotransferase (normal range is 10 U/L to 40 U/L); Scr, serum creatinine (normal range is 50 μmol/L to 100 μmol/L); BUN, blood urea nitrogen (normal range is 3.0 mmol/L to 8.0 mmol/L); BNP, brain natriuretic peptide (normal range is 0 pg/ml to 100 pg/ml); cTn I, cardiac troponin I (normal range is <0.03 ng/ml); CKMB, creatine kinase isoenzyme (normal range is 0.5 ng/ml to 6.0 ng/ml).

Upon arrival in the ICU, the patient was unconscious with a GCS score of 3 (1-1-1), bilateral pupils measuring 4.0 mm with sluggish light reflexes. His heart rate fluctuated between 100 and 120 b/min, and his blood pressure was about 102/66 mmHg. The VA-ECMO was continued for cardiac support. Enteric-coated aspirin (0.1 g, once a day via gastric tube infusion) and clopidogrel (75 mg once daily via gastric tube infusion) were given along with heparin for anticoagulation. Brain natriuretic peptide injection was given to improve cardiac function along with fructose phosphate and trimetazidine for cardiac nutritional support. Continuous ice blanket and ice cap cooling to control the body temperature between 32.0 °C and 34.0 °C was used to reduce the oxygen consumption of brain cells. During hypothermia, the patient received a combination of meperidine hydrochloride and buspirone to manage grade 0–1 chills during hypothermia, as assessed by the bedside shivering assessment scale. Concurrently, albumin (10 g, q8h, intravenous), furosemide (10 mg, q12h, intravenous) and mannitol (250 ml, q8h, intravenous) were used to reduce cerebral edema; this approach was conducive to brain recovery. Continuous renal replacement therapy (CRRT) was connected to the ECMO machine to remove inflammatory substances and maintain internal environment stability. Intravenous glycyrrhizin was administered to treat liver damage and omeprazole to protect the gastric mucosa against stress ulceration. Piperacillin tazobactam was administered for infection prophylaxis. In addition, the patient received parenteral nutrition, including fat emulsions, amino acids, glucose, alanyl-glutamine, and vitamins, and gradually changed over to enteral nutrition treatment.

**Figure 3:**
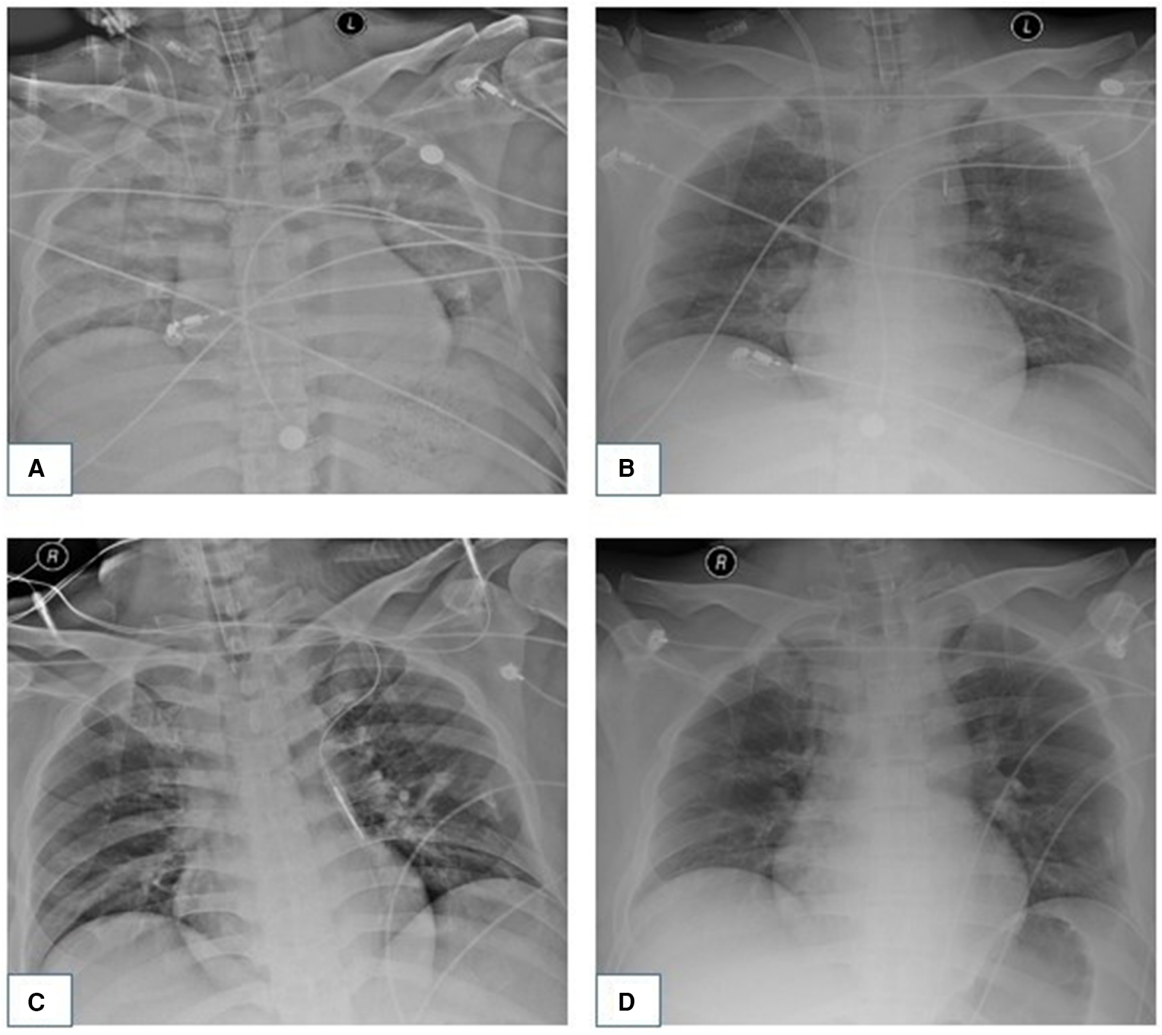
Dynamic changes of the patient's chest x-ray examination. (**A**) On August 18, 2021, chest radiograph showed that bilateral pneumonia and bilateral pulmonary exudative lesions were severe. (**B**) On August 21, 2021, double pneumonia and exudative lesions showed improvement compared with those in the last assessment. (**C**) On August 31, 2021, the patient's chest radiograph showed a significant improvement in double pneumonia. (**D**) The patient's pneumonia had been cured by September 6, 2021.

On August 18, 2021, we obtained a bedside chest radiograph revealing severe exudative lesions in both lungs (Figure 3). On August 19, 2021, the patient's cardiac Doppler ultrasound showed that his EF was 40%, indicating that the cardiac function had improved. The patient's circulation also stabilized, so the vasoactive drugs were gradually reduced and discontinued. On August 20, the patient was still in a coma, with a GCS score of 5 (1-1-3). Bilateral pupils measured 3.0 mm, with normal light reflexes, and the patient showed contractile responses to the painful stimuli. On August 21, his inflammatory markers showed an improving trend, and his urine output was 50–100 ml/h, indicating good renal perfusion, so the CRRT was stopped. At this time, echocardiography showed that the EF had increased to 45%, and his troponin I level was 10.387 ng/ml, indicating that his cardiac function was better than before. Therefore, we removed the ECMO. Figure 3 shows the reexamination of the chest radiograph.

Starting on August 23rd, the patient received primary rehabilitation training daily to promote the recovery of body functions, and magnetic resonance imaging of the brain showed no obvious abnormalities. On August 26, the sputum culture from the patient indicated a multidrug-resistant, carbapenem-resistant *Acinetobacter baumannii* infection, thus prompting a switch to vancomycin for targeted treatment. On August 31, the patient became conscious and was able to open his eyes independently, and we subsequently extubated him. However, the patient's affect was flat, with a mixed aphasia, and reduced computing and memory functions. The muscle strength of both upper limbs was level 2, and that of both lower limbs was level 4. Reevaluation of chest radiography yielded significantly improved results compared with the previous examination (Figure 3). On September 6, the chest radiograph revealed no significant pulmonary exudation (Figure 3), and the patient was transferred from the ICU to the general ward. On September 24, we performed coronary artery bypass grafting for the patient without any complications. The patient was discharged on October 11, 2021. To date, the patient has been recovering well.

## 3 Discussion

CA occurs when the heart stops beating suddenly due to various causes in unexpected circumstances and at unexpected times. This leads to sudden suspension of effective cardiac pump functions and effective circulation, and causes severe ischemia, hypoxia, and metabolic disorders of tissues and cells in the body. If the patient is not resuscitated in time, they will die. Among the causes of sudden CA, the most common are cardiogenic, such as coronary heart disease, cardiomyopathy, acute or chronic heart failure, valvular heart disease, and myocarditis. In addition, pulmonary embolisms, pneumothoraces, electric shock, electrolyte disorders, drowning, rupture of aortic dissections, and severe trauma can also cause CA. In the past, the resuscitation of patients with CA was limited to chest compressions and drug therapy.

In 1953, Gibbon invented the artificial heart-lung machine, which successfully used cardiopulmonary bypass technology during cardiac surgery for the first time, making it possible for the artificial heart-lung machine system to be used for longer times. With the development of this technique, ECMO has been used as a mechanically assisted vital support technique for a variety of cardiac diseases, including cardiogenic shock (6–12), fulminant myocarditis (13), acute coronary syndrome (14, 15), as a bridge for durable mechanical circulatory support or transplantation (16), as an adjunct to cardiopulmonary resuscitation (17, 18), for refractory CA (19), and for primary graft failure and secondary heart graft rejection (20). Its basic principle is to pump the patient's deoxygenated venous blood through a power pump (artificial heart), perform gas exchange through an artificial membranous lung, increase the blood's oxygen content and remove the carbon dioxide, and then pump the blood back into the patient's body to provide adequate tissue perfusion in patients with cardiac function failure. ECMO can replace cardiopulmonary functions for a period of time, providing circulatory and respiratory support for the body and enabling the heart and lungs to rest, creating the opportunity for healing of the heart and lung pathologies.

Although the patient recovered a sinus rhythm following CCPR and repeated defibrillation, the color Doppler echocardiography showed that the patient's heart function was still poor, and the circulation was unstable. Therefore, we immediately implemented VA-ECMO to support the patient's circulatory and respiratory functions and found that the patient's LMCA, anterior descending branch, and circumflex branch were significantly narrowed by performing coronary angiography. After being transferred to the ICU, the patient continued to receive systemic organ support. VA-ECMO played a key role in this patient's management. The implementation of VA-ECMO not only guarantees effective systemic perfusion, but also coronary arteriography can be performed quickly with the support of ECMO to clarify the cause of the CA, and enable timely targeted treatment according to the cause, leading to improved patient prognoses. Of note, the total time of cardiopulmonary resuscitation (CPR) was 144 min when return of spontaneous circulation was achieved. The patient's CA was witnessed, and the time since CA to initiation of CPR by the witness was approximately 2 min for the patient. Although the witness did not have the professional certification of CPR, he had learned some popular science knowledge about CPR. Therefore, popularizing cardiopulmonary resuscitation and adopting high-quality cardiopulmonary resuscitation is conducive for improving the survival rate of patients with OHCA. It is noteworthy that the patient's care took place in 2021 and involved therapeutic hypothermia, in accordance with the 2020 American Heart Association (AHA) guidelines for cardiopulmonary resuscitation and Emergency Cardiovascular Care (21). In contrast, the 2023 European Society of Cardiology (ESC) guidelines for the management of acute coronary syndromes have omitted hypothermia and now advocate solely for active prophylaxis against fever (body temperature >37.7 °C) (22).

## 4 Conclusion

For patients with respiratory and CA, in addition to external cardiac compressions and drug therapy, ECMO can be actively implemented when economic conditions permit. With the support of ECMO, the cause of CA can be identified early, and the treatment can be targeted, thus reducing mortality rates. Promoting cardiopulmonary resuscitation and adopting high-quality cardiopulmonary resuscitation is also conducive for improving the survival rate of patients with OHCA.

## Data Availability

The original contributions presented in the study are included in the article/Supplementary Material, further inquiries can be directed to the corresponding authors.

## References

[1] Shanmugasundaram M Lotun K. Refractory out of hospital cardiac arrest. Curr Cardiol Rev. (2018) 14(2):109–14. 10.2174/1573403X14666180507155622 29737259 PMC6088448

[2] Andersen LW Holmberg MJ Berg KM Donnino MW Granfeldt A. In-hospital cardiac arrest: a review. JAMA. (2019) 321(12):1200–10. 10.1001/jama.2019.1696 30912843 PMC6482460

[3] Zhang S. Sudden cardiac death in China: current status and future perspectives. Europace. (2015) 17(Suppl 2):Ⅱ14–8. 10.1093/europace/euv143 26842111

[4] Thiagarajan RR Barbaro RP Rycus PT Mcmullan DM Conrad SA Fortenberry JD Extracorporeal life support organization registry international report 2016. ASAIO J. (2017) 63(1):60–7. 10.1097/MAT.0000000000000475 27984321

[5] Conrad SA Broman LM Taccone FS Lorusso R Malfertheiner MV Pappalardo F The extracorporeal life support organization Maastricht treaty for Nomenclature in extracorporeal life support. A position paper of the extracorporeal life support organization. Am J Respir Crit Care Med. (2018) 198(4):447–51. 10.1164/rccm.201710-2130CP 29614239 PMC6118026

[6] Bakhtiary F Keller H Dogan S Dzemali O Oezaslan F Meininger D Venoarterial extracorporeal membrane oxygenation for treatment of cardiogenic shock: clinical experiences in 45 adult patients. J Thorac Cardiovasc Surg. (2008) 135:382–8. 10.1016/j.jtcvs.2007.08.007 18242273

[7] Elsharkawy HA Li L Esa WA Sessler DI Bashour CA. Outcome in patients who require venoarterial extracorporeal membrane oxygenation support after cardiac surgery. J Cardiothorac Vasc Anesth. (2010) 24:946–51. 10.1053/j.jvca.2010.03.020 20599396

[8] Hsu PS Chen JL Hong GJ Tsai YT Lin CY Lee CY Extracorporeal membrane oxygenation for refractory cardiogenic shock after cardiac surgery: predictors of early mortality and outcome from 51 adult patients. Eur J Cardiothorac Surg. (2010) 37:328–33. 10.1016/j.ejcts.2009.07.033 19748279

[9] Rastan AJ Dege A Mohr M Doll N Falk V Walther T Early and late outcomes of 517 consecutive adult patients treated with extracorporeal membrane oxygenation for refractory postcardiotomy cardiogenic shock. J Thorac Cardiovasc Surg. (2010) 139:302–11. 311.e1. 10.1016/j.jtcvs.2009.10.043 20106393

[10] Wang J Han J Jia Y Zeng W Shi J Hou X Early and intermediate results of rescue extracorporeal membrane oxygenation in adult cardiogenic shock. Ann Thorac Surg. (2009) 88:1897–903. 10.1016/j.athoracsur.2009.08.009 19932257

[11] Wu MY Lin PJ Lee MY Tsai FC Chu JJ Chang YS Using extracorporeal life support to resuscitate adult postcardiotomy cardiogenic shock: treatment strategies and predictors of short-term and midterm survival. Resuscitation. (2010) 81:1111–6. 10.1016/j.resuscitation.2010.04.031 20627521

[12] Zhang R Kofidis T Kamiya H Shrestha M Tessmann R Haverich A Creatine kinase isoenzyme MB relative index as predictor of mortality on extracorporeal membrane oxygenation support for postcardiotomy cardiogenic shock in adult patients. Eur J Cardiothorac Surg. (2006) 30:617–20. 10.1016/j.ejcts.2006.07.016 16934992

[13] Hsu KH Chi NH Yu HY Wang CH Huang SC Wang SS Extracorporeal membranous oxygenation support for acute fulminant myocarditis: analysis of a single center’s experience. Eur J Cardiothorac Surg. (2011) 40:682–8. 10.1016/j.ejcts.2010.12.050 21334919

[14] Bermudez CA Rocha RV Toyoda Y Zaldonis D Sappington PL Mulukutla S Extracorporeal membrane oxygenation for advanced refractory shock in acute and chronic cardiomyopathy. Ann Thorac Surg. (2011) 92:2125–31. 10.1016/j.athoracsur.2011.07.029 21982150

[15] Kim H Lim SH Hong J Hong YS Lee CJ Jung JH Efficacy of veno-arterial extracorporeal membrane oxygenation in acute myocardial infarction with cardiogenic shock. Resuscitation. (2012) 83:971–5. 10.1016/j.resuscitation.2012.01.037 22322287

[16] Pagani FD Aaronson KD Swaniker F Bartlett RH. The use of extracorporeal life support in adult patients with primary cardiac failure as a bridge to implantable left ventricular assist device. Ann Thorac Surg. (2001) 71:S77–81. 10.1016/s0003-4975(00)02620-5 11265871

[17] Chung SY Sheu JJ Lin YJ Sun CK Chang LT Chen YL Outcome of patients with profound cardiogenic shock after cardiopulmonary resuscitation and prompt extracorporeal membrane oxygenation support. A single-center observational study. Circ J. (2012) 76:1385–92. 10.1253/circj.cj-11-1015 22447007

[18] Wu MY Lee MY Lin CC Chang YS Tsai FC Lin PJ. Resuscitation of non-postcardiotomy cardiogenic shock or cardiac arrest with extracorporeal life support: the role of bridging to intervention. Resuscitation. (2012) 83:976–81. 10.1016/j.resuscitation.2012.01.010 22269099

[19] Massetti M Tasle M Le Page O Deredec R Babatasi G Buklas D Back from irreversibility: extracorporeal life support for prolonged cardiac arrest. Ann Thorac Surg. (2005) 79:178–84. 10.1016/j.athoracsur.2004.06.095 15620939

[20] Kittleson MM Patel JK Moriguchi JD Kawano M Davis S Hage A Heart transplant recipients supported with extracorporeal membrane oxygenation: outcomes from a single-center experience. J Heart Lung Transplant. (2011) 30:1250–6. 10.1016/j.healun.2011.05.006 21676629

[21] Panchal AR Bartos JA Cabañas JG Donnino MW Drennan IR Hirsch KG Adult basic and advanced life support writing group. Part 3: adult basic and advanced life support: 2020 American Heart Association guidelines for cardiopulmonary resuscitation and emergency cardiovascular care. Circulation. (2020) 142(16_suppl_2):S366–468. 10.1161/CIR.0000000000000916 33081529

[22] Byrne RA Rossello X Coughlan JJ Barbato E Berry C Chieffo A ESC Scientific document group. 2023 ESC guidelines for the management of acute coronary syndromes. Eur Heart J. (2023) 44(38):3720–826. 10.1093/eurheartj/ehad191 37622654

